# Deep Learning Algorithms in the Automatic Segmentation of Liver Lesions in Ultrasound Investigations

**DOI:** 10.3390/life12111877

**Published:** 2022-11-14

**Authors:** Mădălin Mămuleanu, Cristiana Marinela Urhuț, Larisa Daniela Săndulescu, Constantin Kamal, Ana-Maria Pătrașcu, Alin Gabriel Ionescu, Mircea-Sebastian Șerbănescu, Costin Teodor Streba

**Affiliations:** 1 Department of Automatic Control and Electronics, University of Craiova, 200585 Craiova, Romania; 2 Oncometrics S.R.L., 200677 Craiova, Romania; 3 Department of Gastroenterology, Emergency County Hospital of Craiova, 200642 Craiova, Romania; 4 Department of Gastroenterology, Research Center of Gastroenterology and Hepatology, University of Medicine and Pharmacy of Craiova, 200349 Craiova, Romania; 5 Department of Pulmonology, University of Medicine and Pharmacy of Craiova, 200349 Craiova, Romania; 6 Department of Hematology, University of Medicine and Pharmacy of Craiova, 200349 Craiova, Romania; 7 Department of History of Medicine, University of Medicine and Pharmacy of Craiova, 200349 Craiova, Romania; 8 Department of Medical Informatics and Statistics, University of Medicine and Pharmacy of Craiova, 200349 Craiova, Romania

**Keywords:** ultrasound, image segmentation, hepatocarcinoma, liver tumor

## Abstract

Background: The ultrasound is one of the most used medical imaging investigations worldwide. It is non-invasive and effective in assessing liver tumors or other types of parenchymal changes. Methods: The aim of the study was to build a deep learning model for image segmentation in ultrasound video investigations. The dataset used in the study was provided by the University of Medicine and Pharmacy Craiova, Romania and contained 50 video examinations from 49 patients. The mean age of the patients in the cohort was 69.57. Regarding presence of a subjacent liver disease, 36.73% had liver cirrhosis and 16.32% had chronic viral hepatitis (5 patients: chronic hepatitis C and 3 patients: chronic hepatitis B). Frames were extracted and cropped from each examination and an expert gastroenterologist labelled the lesions in each frame. After labelling, the labels were exported as binary images. A deep learning segmentation model (U-Net) was trained with focal Tversky loss as a loss function. Two models were obtained with two different sets of parameters for the loss function. The performance metrics observed were intersection over union and recall and precision. Results: Analyzing the intersection over union metric, the first segmentation model obtained performed better compared to the second model: 0.8392 (model 1) vs. 0.7990 (model 2). The inference time for both models was between 32.15 milliseconds and 77.59 milliseconds. Conclusions: Two segmentation models were obtained in the study. The models performed similarly during training and validation. However, one model was trained to focus on hard-to-predict labels. The proposed segmentation models can represent a first step in automatically extracting time-intensity curves from CEUS examinations.

## 1. Introduction

The ultrasound (US) is one of the most used medical imaging investigations worldwide. It is a cheap, safe, and effective modality that can detect a large range of lesions, especially in the case of parenchymatous organs. Therefore, it is especially effective in assessing liver tumors and other types of parenchymal changes; this in turn makes it a prime investigation in the screening of malignancies [1]. This becomes even more important when dealing with at-risk populations, such as patients suffering from hepatitis or cirrhosis of either viral or noninfectious origin. Liver ultrasound is routinely performed by different medical specialties, depending on local regulations and after completing a training course and obtaining the necessary competencies [2]. These training programs vary in length, number of required steps, or addressability. Point-of-care ultrasound (POCUS) has become increasingly utilized worldwide as a screening method, being performed in various medical settings from the emergency room to the general practitioner’s office [3].

However, US has become an indispensable tool when diagnosing liver cancer, mainly due to the application of contrast agents. Safe, reliable, and minimally invasive, contrast-enhanced US (CEUS) can be applied to almost any patient due to virtually non-existent allergic reactions or organ dysfunction that may restrict its usage. The contrasting agent relies on gas microbubbles that are injected intravenously, reach liver vasculature, are degraded intravascularly under ultrasound, the produced gas being harmlessly excreted through the lungs. Liver tumors, especially hepatocellular carcinoma (HCC) produce specific filling patterns during CEUS [4]. The physician relies on following a fixed region containing the lesion over a long period of time and studying the contrast uptake and later wash-out pattern, thus indicating the possible diagnosis. The interpretation of CEUS strongly relies on the experience of the performing physician [5]. One reliable method to quantify contrast uptake and, subsequently, describe tumor vasculature, is the generation of time intensity curves (TIC) that can be later analyzed, providing a diagnosis of malignancy [6].

Multiple artifacts pertaining to breathing motion or movements of the US probe may degrade the quality of TICs generated during a normal investigation of the liver. Artificial intelligence can be employed to segment the liver parenchyma in normal B-mode US to maintain a stable area of interest (AOI) around the focal liver lesion that needs characterization. The procedure to manually adjust the AOI is time-consuming and subject to multiple sources of error [7].

Image segmentation is a computer vision technique in which each pixel from an image has a specific class associated with it. Depending on the purpose of the segmentation, the output image can have two classes (binary image) or more than two classes. In the field of medical imaging, both types of image segmentation techniques are used. Two class segmentation (binary images) is used as a mask to extract pathological or physiological information about a patient [8]. In medical imaging, multi-class image segmentation (semantic segmentation) is used to classify and extract different types of lesions [9]. In the last years there have been many solutions proposed for medical image segmentation based on artificial intelligence (AI) algorithms. These solutions can be classified as machine learning (ML) approaches and deep learning (DL) approaches. Several ML algorithms are used for image segmentation, among them are k-means and fuzzy c-means [10]. As for DL algorithms, several models based on convolutional neural networks were proposed for medical imaging segmentation. The U-Net model proposed by Olaf Ronneberger et al. [11] is a fully convolutional neural network for biomedical image segmentation. Other models were derived from the U-Net model, such as V-Net, proposed by Fausto Milletari et al. [12], are used for volumetric medical segmentation. Attention U-Net proposed by Ozan Oktay et al. [13] is used on medical image segmentation to focus on target structures. Another derived work from U-Net is U-Net++, proposed by Zongwei Zhou et al. [14], which is a nested U-Net architecture tested in medical image segmentation tasks such as nuclei segmentation, polyp segmentation, and liver segmentation. For the segmentation of the liver and liver lesions in magnetic resonance imaging (MRI) and computer tomography (CT) different authors have proposed methods based on ML or DL algorithms. Jose Denes Lima Araújo et al. [15] proposed a segmentation of liver from CT images using a U-Net model. Sebastian Nowak et al. [16] proposed a method for detecting liver cirrhosis in T2-weighted MRI scans. Their pipeline contained two DL models, one with the goal of performing liver segmentation on the T2-weigthed MRI image and the second with the goal of classifying the segmented image. To date, there are not many works concerning liver lesions segmentation in ultrasound (US) investigations using DL or ML algorithms. The segmentation of the liver lesions in US is a challenging task due to interference noise and missing boundaries of lesions [17]. In their work, Nishan Jain et al. [18] proposed a method for US liver segmentation using region difference filters. In their method, four different filters were applied to the image obtaining a region-difference image. A pixel was manually selected from the lesion. The resulting mask was computed by transforming the region-difference image into a binary image and the ROI was considered as the nearest edges which enclosed the selected pixel. Their dataset contained 56 B-mode ultrasound investigations. In terms of performance metrics, the average accuracy was measured in their study, obtaining values from 65.2 to 99.68%. Deep Gupta et. al. [19] proposed a hybrid segmentation method for US images. The method was based on the Gaussian kernel-based fuzzy c-means clustering algorithm and region based active contour model. Based on the authors conclusions, their method provided better accuracy compared with other methods such as fuzzy c-means clustering or geodesic active contours. Their dataset contained 50 US investigations. However, the US images did not contain only liver investigations.

Our aim was to build a deep learning model for automatic, real-time image segmentation during US examinations to open new opportunities in automatically computed time-intensity curves. The obtained model should have a maximum inference time of 100 milliseconds to be a baseline for creating a system to perform liver lesions classification in contrast-enhanced ultrasound investigations. Thus, besides evaluating the model in terms of performance metrics, the average inference time was also observed on two different environments. As mentioned earlier, US has become an indispensable tool when diagnosing liver cancer. It is cheap, non-invasive and can detect different types of lesions. However, US investigations have noise which can affect the interpretation of the results. A DL model which can perform image segmentation of liver lesions can be a useful tool for a gastroenterologist, especially when extracting time-intensity curves. Compared to the studies mentioned earlier, our proposed method used a DL model for the segmentation of liver lesions. More specifically, the architecture of the DL model is U-Net [11]. U-Net is an “U” shaped DL model architecture with two connected parts: the encoder and the decoder. Due to this architecture, U-Net required a single run on an image to predict the mask.

## 2. Materials and Methods

### 2.1. Data Acquisition

The dataset used in our study was provided by the University of Medicine and Pharmacy Craiova, Romania. The contrast-enhanced ultrasound examinations were performed using a Hitachi Aloka Arietta V70 350 (Hitachi Medical Corporation, Tokyo, Japan), equipped with the convex probe C 251 (Hitachi Medical Corporation, Tokyo, Japan). For CEUS, the contrast agent used was SonoVue (Bracco SpA, Milano, Italia). The dataset contained 50 video files from 49 consecutive patients with 59 liver lesions. Each video file was in audio video interleave (AVI) format encoded with motion jpeg and with a bit rate of 31,196 kb/s. The video files had between 7 and 12 frames per second. The video files did not contain the entire examination, they were divided into three phases: arterial, portal venous, and late phase. However, it was not an impediment as in this stage since each video file was treated individually without the need to know which file belongs to which patient and what lesion type an image contains. A more detailed analysis of the dataset is documented in [20].

### 2.2. Data Preprocessing

Before extracting frames from the video files, the region in B-mode was defined for each type of ultrasound device. This region was defined to automatically crop and extract frames from the video examinations in the dataset. Table 1 contains these regions for all the ultrasound devices used in this study. The values for x and y axis were determined experimentally by plotting the frame and checking the coordinates for B-mode. 

**Figure 1:**
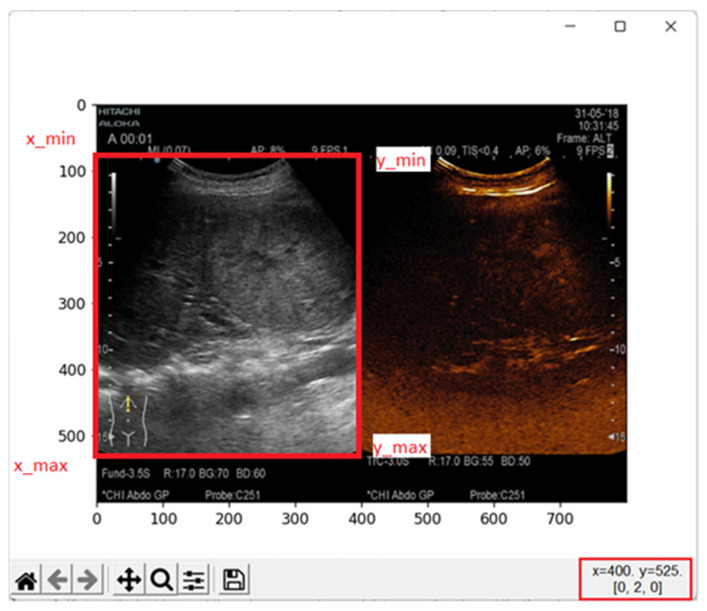
Plotting the first frame of the video examination and determining the edges of the B-mode image. Bottom right coordinate presented in red box (X max and Y max).

Figure 1 shows how these regions were determined. After the x and y coordinates were determined for each ultrasound device, the next step was frame extraction. As mentioned, each video examination had between 7 and 12 frames per second. The changes of lesion in terms of pixels from one second to another were relatively small, therefore we have extracted the frames with a sample time of 1 s. Extracting and marking all the frames from each video examination would have resulted in marking almost the same image multiple times. Algorithm 1, used for extracting the frames from the videos, is presented below.
**Algorithm 1.** Extracting frames from the video examinationfps ← 0 ultrasound_device ← ultrasound_device_characteristics_object fps ← ultrasound_device.getFPS() frame_height ← read frame height of the video examination frame_width ← read frame width of the video examination b_mode_x_min ← ultrasound_device.b_mode_x_min b_mode_x_max ← ultrasound_device.b_mode_x_max b_mode_y_min ← ultrasound_device.b_mode_y_min b_mode_y_max ← ultrasound_device.b_mode_y_max**while** video examination file still has frames **do**:  frame_id ← get the frame id from the video file  frame ← get the frame from the video file  **if** frame_id % fps == 0 **do**:      //Proccess the frame and save it to disk.      Cropped_frame ← frame[b_mode_x_min: b_mode_x_max,          b_mode_y_min, b_mode_y_max]      save cropped_frame to disk  **else**:      continue //ignoring the current frame

**Figure 2:**
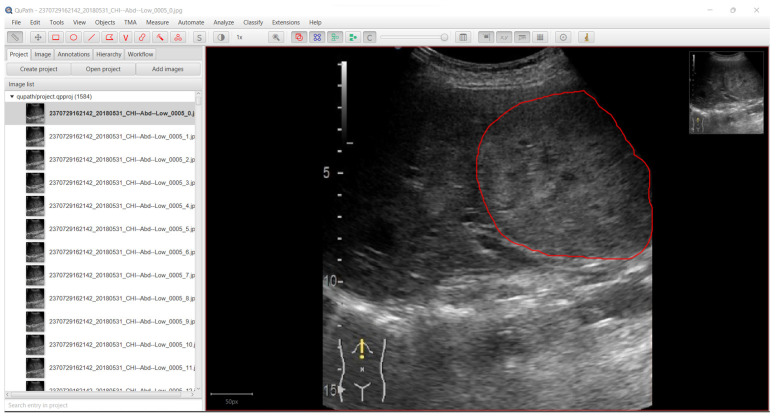
QuPath, the software used to mark the lesions and to apply Algorithm 2 for mask generation.

**Figure 3:**
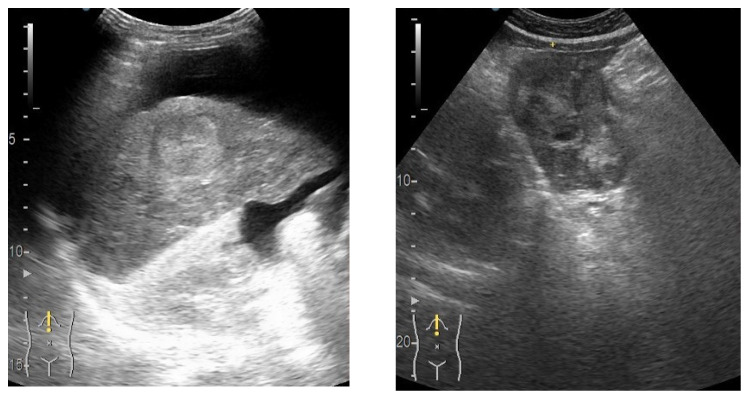
Example of frames extracted by Algorithm 1.

Extracting the frames from each video resulted in a total number of 6035 image files in B-mode. The region of interest (ROI) for each image was marked by a senior gastroenterologist (L.D.S.) with over 20 years of experience in abdominal ultrasound interpretation. For annotation of the region of the interest, QuPath software was used [21]. Figure 2 shows a screen capture from QuPath with a liver lesion marked by the expert gastroenterologist (L.D.S.). Examples of frames extracted by Algorithm 1 are presented in Figure 3.

**Figure 4:**
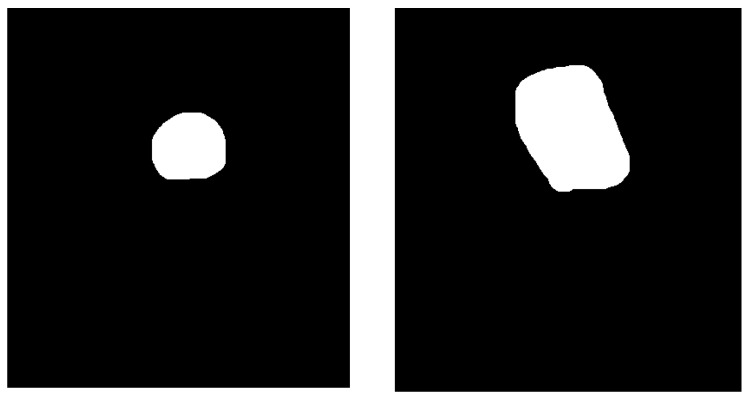
Example of masks obtained from Algorithm 2.

For developing the algorithm, for each image in B-mode the corresponding label had to be created. The label was defined as a binary image with the same size as the input image. As shown in Figure 2, each lesion was marked as a shape on top of the B-mode cropped image. After every lesion was marked by the expert gastroenterologist, in order to export the binary images (masks) from QuPath [21], Algorithm 2 was applied for every image in the project. Example of masks obtained from Algorithm 2 are presented in Figure 4. The masks presented in Figure 4 were obtained by running the frames presented as an example in Figure 3.
**Algorithm 2.** Mask creation (binary image)current_image ← obtain current image **while** current_image is not null **do**:   mask ← new Image(current_image.width, current_image.height, values = 0)      **for each** object in annotation_list **do**:         roi ← object.getROI()         roi.fill(values = 1)         mask ← mask **bitwise and** roi   mask_filename ← string concatenation (current_image.name, “-mask”)   save image to disk (mask, mask_filename)


**Figure 5:**
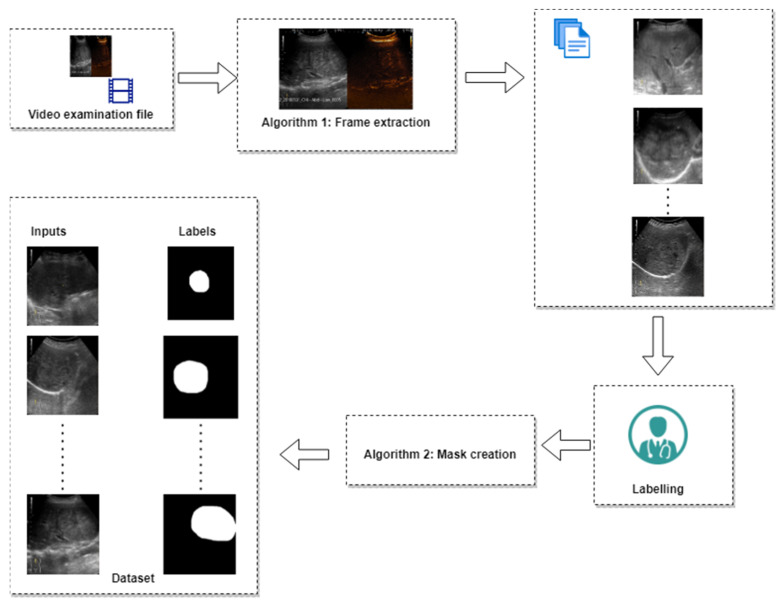
Dataset preparation pipeline.

After data preprocessing, 6035 images in B-mode were obtained (with 6035 corresponding masks). The dataset preparation pipeline is presented in Figure 5. The video examination file was passed through Algorithm 1 to extract the frames. The sample time used was 1 second. The frames obtained were labelled and then each frame was passed through Algorithm 2 to obtain the mask (a binary image in which the black color represented normal tissue and the white color represented the lesion(s)). Algorithm 1 was implemented in Python programming language since the main goal of the study was to perform real-time image segmentation. Thus, Algorithm 1 will be reused in further developments. On the other hand, Algorithm 2 was developed using QuPath Automate function. The QuPath script editor (Automate) allowed us to process the images in the current project in batch. Hence, Algorithm 2 was implemented in QuPath to easily export the masks created by the expert gastroenterologist L.D.S. (Figure 2).

### 2.3. Neural Network Model

In our study, the goal of the deep learning model was to perform semantic segmentation to each frame of the video examination to proper identify the lesion location. To accomplish this, we have trained a U-Net model proposed by Olaf Ronneberger et al. [11] with an input size of 256 by 256 pixels. All the images and their corresponding masks were resized (without locking aspect ratio) to fit the proposed model. U-Net is an “U” shaped fully convolutional network. The architecture has a down sampling branch (encoder) and an up-sampling branch (decoder). U-Net and other versions derived from it are used in many applications for biomedical imaging segmentation like lung ultrasound segmentation [22], cardiac magnetic resonance imaging segmentation [23], bone segmentation [24], or bones lesions segmentation [25]. Due to its architecture, U-Net can extract discriminative features from the raw images with limited training data. Segmentation of a 512 by 512 image takes less than a second [11]. The input in the U-Net architecture used in the study was a tensor with a size of 256 by 256 pixels. The encoder part of the model contained 4 blocks of convolutional layers. Each block applied two convolutional filters with a kernel size of 3 by 3 pixels and no padding. After each block of convolutional layers, a max pooling operation was performed with size 2 × 2 and stride 2. This operation was performed for down sampling the input tensor. The first convolutional block had 64 filters. The number of filters doubled after each max pooling operation. The scope of the encoder part of the model was to decrease resolution and increase depth to capture the context. The activation function in the encoder part for all the convolutional layers was a rectified linear unit (ReLu). The decoder part of the model contained 4 blocks of convolutional layers. Similarly to the encoder part, it applied two convolutional filters with a kernel size of 3 by 3 pixels without padding. After each block of convolutional layers, an up-sampling operation with kernel size 2 × 2 and nearest neighbor interpolation was performed. The resulting model had a total of 412,865 trainable parameters from a total of 414,401.

### 2.4. Hyperparameters and Loss Function

**Figure 6:**
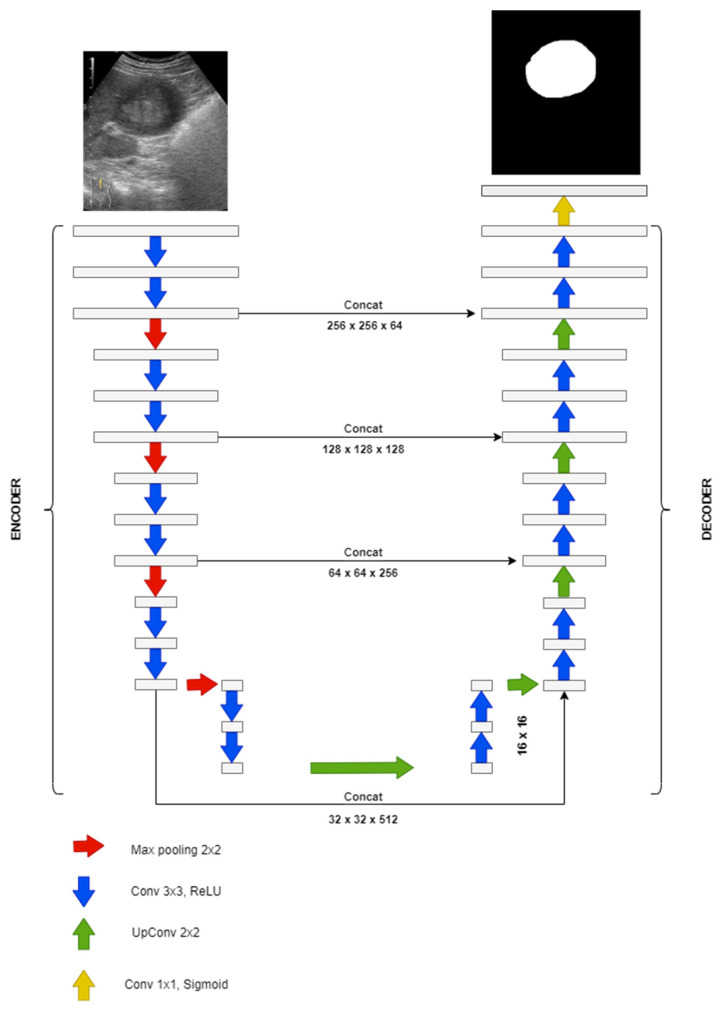
Architecture of the proposed segmentation model.

When training a neural network, the dataset should be split into training data, validation data, and testing data. The training and validation groups are used during training while the testing group it is used for testing the neural network. For the proposed model, we randomly divided the dataset as follows: 70% for training, 20% for validation, and 10% for testing. The model was trained for 25 epochs with a batch size of 8. The optimizer used for the model was Adam, an optimization algorithm with adaptive learning rate created specifically for training neural networks. The optimizer is computationally efficient since it can find individual learning rates for each parameter. It requires the following inputs: alpha (α)—the learning rate or step size, beta (β_1_) and beta2 (β_2_)—the exponential decay rates for the moment estimates and a very small number, and epsilon (ε), to prevent division by zero [26]. The values we have chosen for these inputs are presented in Table 2. For beta1, beta2, and epsilon the values recommended in [26] were used. The architecture of the proposed segmentation mode is presented in Figure 6.

Since the goal of the proposed model was to predict whether a certain pixel in an ultrasound image belonged to a liver lesion or not, it can be assumed that the model was a binary classifier. Typically, in a binary classification task, the loss function used is binary cross entropy (BCE). Cross entropy is a method to measure the difference between two probability distributions and it is given by Equation (1).

(1) $$CE=-\sum_{i=1}^{C}t_{i\;}\log\left(s_{o,i}\right)$$

where *t_i_* is the truth label, *s_o,_**_i_*** is the predicted probability observation, *o* is of class *i*, and *C* is the total number of labels. In binary classification, the total number of classes is 2. Replacing C in Equation (1) we obtain (2). In binary cross entropy, when the predicted probability approaches 1, the loss decreases, and when the predicted value is decreasing, the loss starts to increase very fast. From Equation (2) it can be concluded that binary cross entropy penalizes equally for each class. For the proposed image segmentation model, the classes were not balanced, meaning that the pixels with lesions and the pixels with no lesions were not equally distributed into an image. Hence, for the proposed model, the loss function used was focal Tversky loss [16]. The loss function is given by Equations (3) and (4). The model was trained with two sets of α, β, and γ as described in Table 3.

(2) BCE=−(ylog(p)+(1−y)(1−p))

Tversky loss, also known as Tversky index (*TI*) [27] is an asymmetric similarity function (3) in which *X*\*Y* and *Y*\*X* represent the set difference of *X* and *Y*. α and β are two parameters of Tversky index and they must be greater than or equal to 0. If α and β both have the same value, more precisely 0.5, the Tversky index represents the dice coefficient [28]. In contrast, if α and β are set to 1, the Tversky index represents the Jaccard Index, also known as intersection over union (IoU). The Tversky index allows the proper balance of the false positives and false negatives. During training, TI can have serious problems in segmenting small liver regions since these regions do not have a major contribution to the loss. Focal loss [29] solves this problem by adding a factor to the *TI* to focus more on inputs that are hard to segment. Focal Tversky loss (*FTL*) is given by (4). γ is called a focusing parameter and it adjusts smoothly how the easy-to-segment inputs are down weighted [29].

(3) $$TI\left(X,Y\right)=\frac{\left|X\;\cap^{\;}Y\right|}{\left|X\;\cap^{\;}Y\right|+\alpha \left|X\;\backslash Y\right|+\beta \left|Y\backslash X\right|}$$

(4) $$FTL={\left(1-TI\right)}^{\gamma }\;$$

### 2.5. Experimental Setup

All the implementations were performed in Python version 3.9.0 with TensorFlow version 2.7.0 [30] on a machine equipped with Intel i7 Processor, 16 GB RAM, and Nvidia 3050Ti GPU. The operating system used was Windows 11 Pro.

### 2.6. Assessing the Performance of the Deep Neural Network Model

Using only pixel accuracy to measure the performance of the model can be misleading when evaluating the entire system. Assuming that a lesion in a frame from a US examination is only 10% of the entire image and the measured accuracy for that specific image is 90%, it can be concluded that the model is performing well. However, if only the non-lesion pixels were classified correctly, the model was not evaluated correctly. The issue of class imbalance in training deep learning algorithms can be corrected by applying oversampling techniques [31]. However, image segmentation scenarios, in which the liver tissue dominates the image or vice-versa, can be solved by observing more complex and precise metrics that deals with class imbalance. To properly assess the performance of the model, the metrics measured are intersection over-union (IoU or Jaccard index), area under curve (AUC), recall, and precision. Assuming that *TP*, *TN*, *FP*, and *FN* are true positives, true negatives, false positives, and false negatives, respectively, the metrics are defined by Equations (5)–(7). IoU or Jaccard index represents the overlap between model predictions and imagist segmentation divided by the union between model predictions and imagist segmentation. This metric is very useful in image segmentation to assess how much of the predicted mask overlaps with the mask created by the expert gastroenterologist.

(5) $$IoU=\frac{TP}{TP+FP+FN}$$

(6) $$Recall=\frac{TP}{TP+FN}$$

(7) $$Precision=\frac{TP}{TP+FP}$$

(8) $$Dice=\frac{2TP}{2TP+FP+FN}\;$$

(9) $$IoU=\frac{Dice}{2-Dice}$$

## 3. Results

We have built two models with the same architecture and hyperparameters; the only difference between these models were the α, β, and γ inputs for the FTL. The first model, for which the FTL had α equal with β equal with 0.5, did not focus on difficult-to-predict masks.

**Figure 7:**
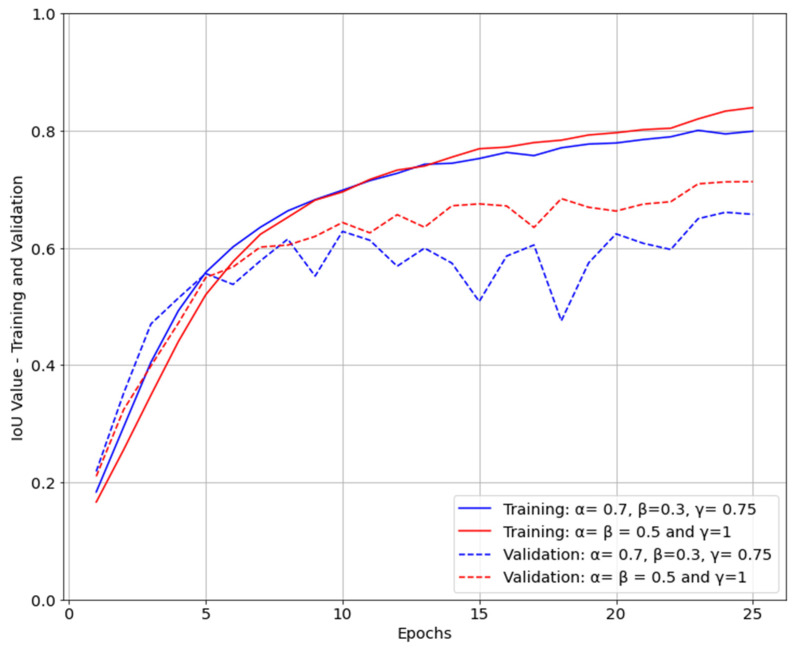
IoU during training and validation.

**Figure 8:**
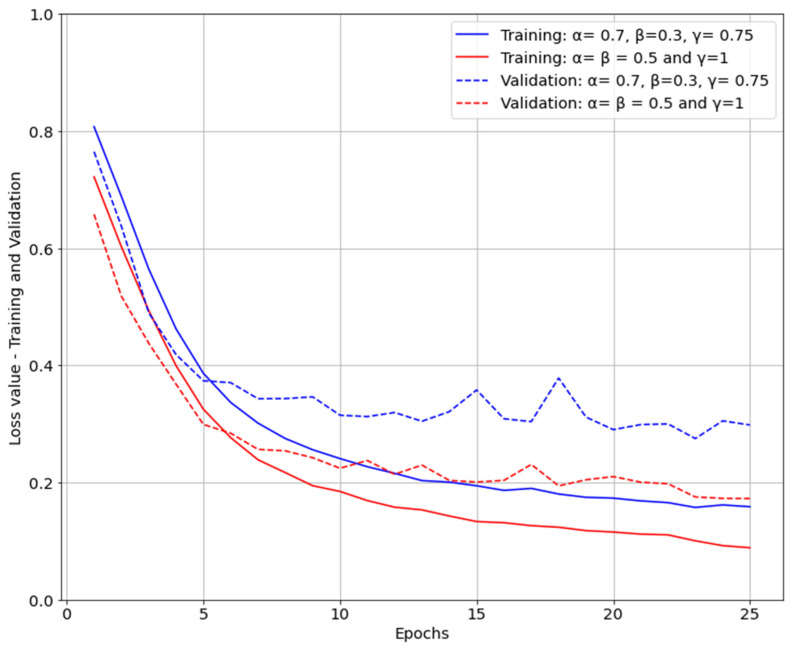
Loss evolution during training and validation.

It can be observed from Table 3 that the U-Net segmentation model trained with parameters α=β=0.5 and γ=1 performed better compared to the model trained with focus on masks which were difficult to predict, α = 0.7 and β = 0.3 γ = 0.75. When loss function of the model was dice coefficient (α=β=0.5), both loss and IoU were converging faster compared with the model with parameters α = 0.7, β = 0.3 γ = 0.75 for FTL (Figure 7 and Figure 8). Furthermore, the IoU obtained for the two models were slightly different, with 0.8392 for the first model and 0.7990 for the second model. However, the first model was performing equally for both easy- and hard-to-predict inputs while the second model was performing relatively poorly on easy-to-predict inputs.

**Figure 9:**
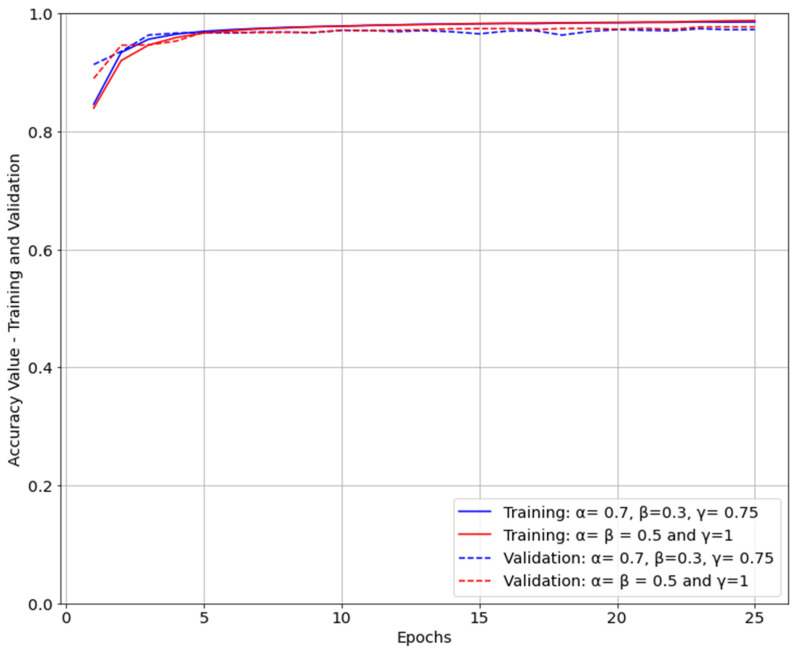
Accuracy evolution during training and validation.

As for evaluating each model, the evolution of accuracy during training was plotted. Figure 9 shows the accuracy of both models during training and validation. It can be observed that the accuracy converged fast. While accuracy has similar values for both models, they were performing very differently, proving that accuracy is not enough to evaluate the performance of a segmentation model.

Besides analyzing the performance of the models in terms of quality of the outputs, an analysis of the inference time, GPU memory utilization, floating point operations (FLOPs), and number of model parameters was carried out. The results were obtained on two different environments. The first test environment (ENV1) was configured on the same machine on which the segmentation models were trained: Intel i7 Processor, 16 GB RAM, and Nvidia 3050Ti GPU. The second test environment was run in the cloud using Google Colab [32]. The virtual machine provided in the Google Colab Jupyter notebook used for testing was configured as follows: Intel Xeon CPU at 2.00 GHz, 12 GB of RAM, and Nvidia Tesla T4 GPU. For testing the inference time, a clean Jupyter Kernel was used. Therefore, before running each test the runtime was restarted. From the entire dataset, 100 frames were randomly selected for testing. Each model was run on each of the selected frames in both test environments. Inference time was measured for both models for all the selected frames. Loading the model into the GPU was measured separately as this action was performed once per each test and it did not influence the inference time of the models. If the models were deployed into a system used in production, loading the model was performed only at the system startup. The metrics measured were minimum inference time, maximum inference time, and average inference time. The results are presented in Table 4 and Table 5.

For analyzing the GPU memory utilization, FLOPs, and total number of model parameters, only ENV1 was used as these metrics were not GPU dependent. The results obtained are presented in Table 6.

## 4. Discussion

The goal of the proposed study was to build a deep learning model which can perform image segmentation from CEUS video examinations. To train and validate the DL segmentation model, we used 59 liver tumors corresponding to 49 patients evaluated through ultrasound and contrast-enhanced ultrasound in the Gastroenterology Department of the Emergency Clinical County Hospital of Craiova between 7 January 2018 and 12 December 2020. Clinical data regarding age, gender, underlying liver disease, history of previous malignancy, the final diagnosis, and the standard method used for confirmation of the diagnosis were collected from the medical records of the patients found in the hospital system. Ultrasound investigations were performed using Hitachi Arietta V 70, convex probe C250. The contrast agent used for the examination was SonoVue (Braco, Milan, Italy). CEUS examinations were stored as videos corresponding to arterial, portal, and delayed phases and were performed according to the European Federation of Societies for Ultrasound in Medicine and Biology (EFSUMB) guidelines. The majority of patients were male, 31 in total (63.26%). The mean age of the patients was 69.57 ± 10.65. In what concerns the presence of a subjacent liver disease, 36.73% had liver cirrhosis and 16.32% had chronic viral hepatitis (5 patients: chronic hepatitis C and 3 patients: chronic hepatitis B). History of previous neoplasia was detected in 11 patients (22.44%). The mean size of the tumors was 51.63 mm. The definitive diagnosis of liver tumors was hepatic hemangioma (n = 6; 10.16%), focal nodular hyperplasia (n = 1; 1.69%), liver cysts (n = 5; 8.47%), liver abscess (n = 1; 1.69%), liver adenoma (n = 1; 1.69%); hepatocellular carcinoma (n = 24; 40.67%), cholangiocarcinoma (n = 4; 6.77%), liver metastases (n = 15; 25.42%), and malignant liver adenoma (n = 1; 1.69%). The final diagnosis was established by contrast-enhanced CT, MRI, or both. CEUS alone was used for typical liver hemangioma or hepatic metastases developed in a known clinical context of neoplasia. The reference diagnosis for uncertain cases was obtained through pathology. A statistical overview of the liver lesions and patients involved in the study is presented in Table 7.

**Figure 10:**
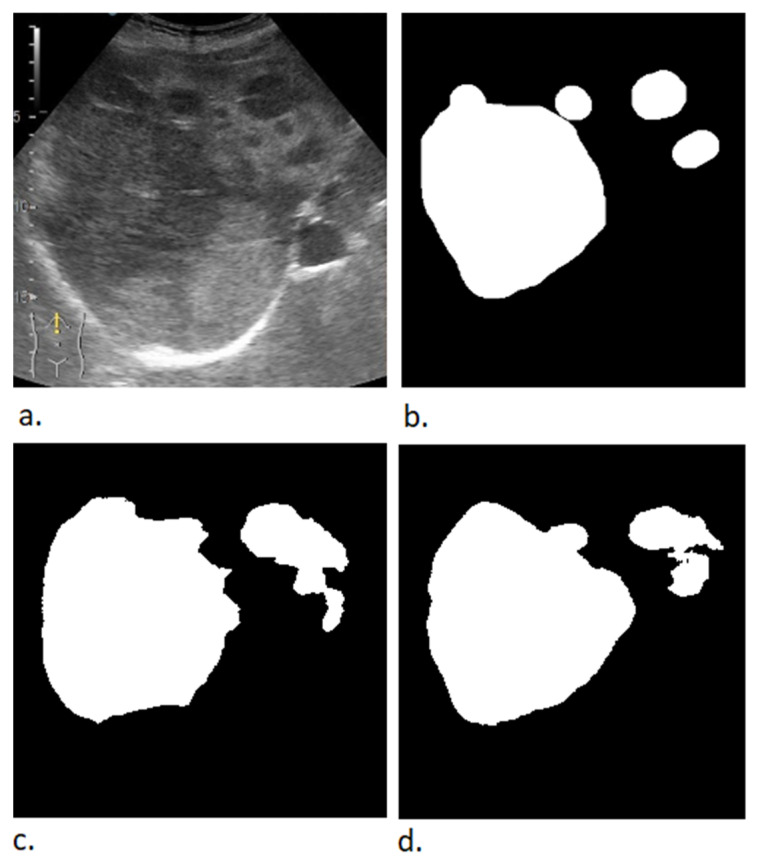
Results from the validation batch. (**a**) B-mode frame from the video examination file. (**b**) Label (expected output). (**c**) Predicted output for model trained with α = β = 0.5 and γ = 1. (**d**) Predicted output for model trained with α = 0.7, β = 0.3, γ = 0.75.

In Figure 10, an example of images from the validation batch is presented. The same video frame was run through both models. Figure 10c presents the predicted output for the model in which the values for the loss function were α = β = 0.5 and γ = 1. Figure 10d presents the predicted output for model in which the values for the loss function were α = 0.7, β = 0.3, and γ = 0.75. The image contains multiple liver lesions with different shapes and sizes. It can be observed that when the lesions are multiple and close to each other, both models predict a much larger region without separation. Moreover, in the predicted mask (Figure 10c) the multiple lesions are not the same shape as marked by L.D.S. and, if a binary operation was performed on the B-mode image with this mask, parenchyma would also be extracted from the image. However, the second model (α = 0.7, β = 0.3, γ = 0.75) is closer in obtaining a separation of the lesions. This shows the fact that during training and validation this model focused on hard-to-predict masks while the other model (α = β = 0.5 and γ = 1 for FTL) treated all the labels from the dataset equally.

**Figure 11:**
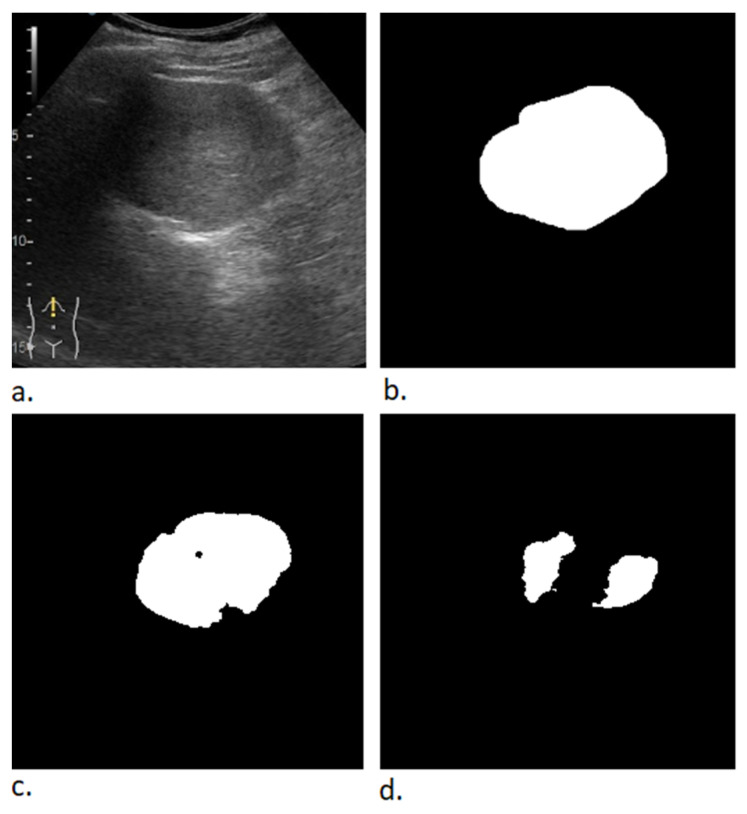
Results from the validation batch. (**a**) B-mode frame from the video examination file. (**b**) Label (expected output). (**c**) Predicted output for model trained with α = β = 0.5 and γ = 1. (**d**) Predicted output for model trained with α = 0.7, β = 0.3, γ= 0.75.

**Figure 12:**
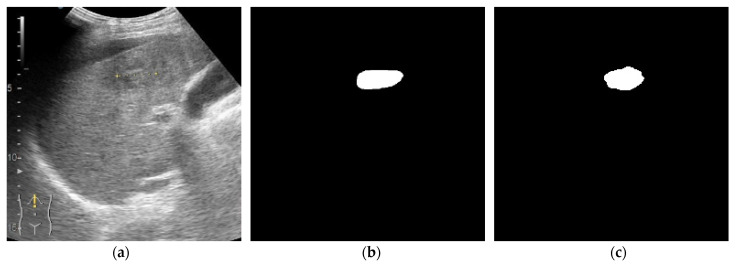
Small tumor size. Results from the validation batch for model with α = β = 0.5 and γ = 1. (**a**) B-mode frame from the video examination file. (**b**) Label. (**c**) Predicted output.

**Figure 13:**
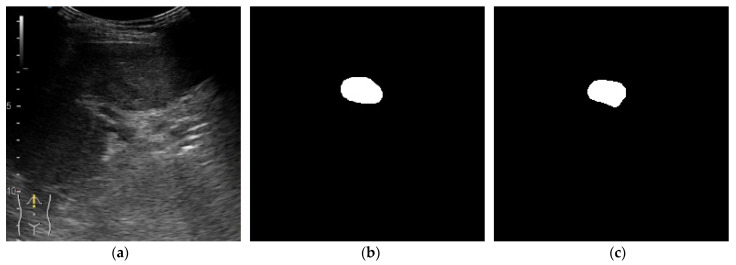
Small tumor size. Results from the validation batch for model with α = 0.7, β = 0.3, γ = 0.75. (**a**) B-mode frame from the video examination file. (**b**) Label. (**c**) Predicted output.

In Figure 11, another image from the validation batch is presented. The lesion is large with no other small lesions in the surrounding tissue. It can be observed that the location of the lesion was predicted correctly by both models, however one model predicted a mask very close to the expected output while the other model predicted two separated lesions. Even though the mean tumor size was 51.65 mm, both obtained segmentation models performed very well on small lesions (Figure 12 and Figure 13).

Other studies [33,34] use dice coefficient [28] for evaluation of image segmentation models. Dice coefficient can be expressed as Equation (8). However, Jaccard index (IoU) can be expressed as related to dice coefficient as in Equation (9) hence observing both metrics when evaluating a segmentation model does not add additional information [35]. There are not many studies which have explored liver tumor segmentation in US. Compared with other existing studies [17,18] in which the authors performed segmentation using region difference filters or fuzzy clustering algorithms, our proposed method used a DL model for the image segmentation of US liver lesions. The architecture used by our study is U-Net [11], a DL model architecture containing two parts: an encoder and a decoder. Due to this architecture, the model requires a single run on an image to predict the corresponding mask. Another advantage of the U-Net model is that it can extract discriminative features from the raw images with limited training data [11]. The proposed method is uses a different approach in terms of loss function and performance metrics observed. Therefore, FTL function was used. As mentioned earlier, observing only accuracy is not enough to assess the performance of a segmentation model. The evolution of accuracy during training and validation for both models is observed in Figure 7. Thus, besides recall and precision, intersection over union was observed for both models. A solution for image segmentation in US investigations based on DL models can be more flexible as transfer learning can be applied on the pre-existing weights of the model. Using transfer learning, the model can be retrained when new datasets are available. Furthermore, a DL model can be trained in a federated learning approach to preserve the privacy of the dataset [36].

As presented in Table 4 and Table 5, the model was tested on two different environments and the results obtained show that the minimum inference time was 32.15 milliseconds and maximum inference time was 77.59 milliseconds. While there is a significant difference between the minimum and maximum inference time, these values are still in the accepted range of a 7 to 12 frames per second video investigation. The memory requirement for the model, presented in Table 6, is 0.9291 GB which is an acceptable size for any modern GPU.

Automatic tumor segmentation in US images is of great importance when using TICs as a method to quantify tumor perfusion in cases of possible liver hepatocellular carcinoma [7]. Keeping the region of interest in focus sometimes proves to be a difficult task, eminently operator-dependent and greatly influenced by comorbidities of the patient (breathing difficulties due to either concomitant respiratory or cardiac diseases, increased abdominal pressure due to underlying cirrhosis, etc.) [37]. In addition, the medical professional tries to keep contrast bubble destruction to a minimum, to maximize the efficiency of the procedure, therefore intentionally moving the probe and thus shifting focus from the liver tumor. The main strength of our method is therefore the capacity to identify the lesion in the regular B-mode window of an US machine, thus providing an accurate TIC measurement for the tumor zone, that can be compared with the one generated by the parenchyma. A possible limitation of our study is the limited number of patients, CEUS examinations and the relatively low variety of tumor types. Furthermore, as presented in Table 4, a possible drawback of the study is the loading time of the model which is 294.29 seconds on ENV1. In image segmentation, the model searches for patterns in B-mode images, and in some cases the lesion shape is more important than the lesion type. If the segmentation model is used for time-intensity curve extraction, a filtering of the values needs to be carried out if sudden changes are observed.

## 5. Conclusions

The aim of the study was to build a DL model for automatic, real-time image segmentation during US examinations. Besides the DL model, two algorithms were defined to achieve the goal. Algorithm 2 was used only for the creation of the dataset while Algorithm 1 was defined to serve two different scopes: dataset preparation and real-time frame extraction and cropping. The DL model used for image segmentation was U-Net with an input of 256 by 256 pixels. The dataset contained 50 video examinations from 49 patients. Two different models were obtained during the study. One model was trained to focus on hard-to-predict labels while the other one was trained to treat all the labels equally. The results presented show that the models built in this study can be useful for gastroenterologists to keep track of lesion movement during examinations. Usually, the extraction of the TIC(s) is performed manually. The operator defines the two regions, lesion and parenchyma, and updates these regions during the entire investigation as the image is moving due to patient’s breathing or if the probe is moving. The inference time was tested for both models in two different environments. The results show that the proposed method can be used for real-time segmentation of US examinations if the video investigations are in a range of 7 to 12 frames per second.

The proposed segmentation models can represent a first step in automatically extracting time-intensity curves from CEUS examinations.

## Figures and Tables

**Table 1:** Coordinates for the edge of B-mode image.

X Min	X Max	Y Min	Y Max
0	400	78	525

**Table 2:** Adam optimizer parameters.

α	β_1_	β_2_	ε
0.0001	0.9	0.999	10^−8^

**Table 3:** Model performance results during training and validation.

Parameters	IoU	Recall	Precision
α=β=0.5 (Dice coefficient) γ=1 (Training/Validation)	0.8392/0.7129	0.8911/0.8256	0.9334/0.8448
α=0.7, β=0.3 γ= 0.75 (Training/Validation)	0.7990/0.6572	0.8171/0.7735	0.9635/0.8192

**Table 4:** Inference time results for ENV1.

Model	Minimum Inference (Milliseconds)	Maximum Inference (Milliseconds)	Average Inference (Milliseconds)	Loading Time (Seconds)
α=β=0.5 (Dice coefficient) γ=1	32.50	56.48	41.76	294.29
α=0.7, β=0.3 γ= 0.75	32.15	59.70	43.04	373.16

**Table 5:** Inference time results for ENV2.

Model	Minimum Inference (Milliseconds)	Maximum Inference (Milliseconds)	Average Inference (Milliseconds)	Loading Time (Seconds)
α=β=0.5 (Dice coefficient) γ=1	48.76	77.59	59.68	5.86
α=0.7, β=0.3 γ= 0.75	51.90	76.43	61.15	7.89

**Table 6:** The complexity of the system.

Metric	Value	Unit
FLOPs	43.2433	MFLOPs
Memory requirement (GPU)	0.9291	GB
Total number of parameters	414,401	N/A

**Table 7:** Patient cohort involved in the study.

Variable	n ^1^ (%)
Gender	M-63.26% F-36.74%
Age (mean value ± SD)	69.57 ± 10.65
Age Wise Classification of Samples	
Age group	Number of patients
<40	2
40–49	2
50–59	7
60–69	17
70+	21
Underlying liver disease	
1. Liver cirrhosis	36.73%
2. Chronic viral hepatitis	HBV-6.12% HCV-10.20%
History of previous malignancy	22.44%
Tumor size (mm), mean value	51.65
Final diagnosis	
Hepatic hemangioma	10.16%
Liver cysts	8.47%
Focal nodular hyperplasia	1.69%
Liver adenoma	1.69%
Liver abscess	1.69%
Hepatocellular carcinoma	40.67%
Liver metastases	25.42%
Cholangiocarcinoma	6.77%
Malignant liver adenoma	1.69%

^1^ n = 49.

## Data Availability

The data presented in this study are available on request from the corresponding author. The images were made available from the University’s repository as an anonymized dataset.
